# 1483. Real-time Creation of HCV Treatment Cascades in Clinics to Improve HCV Treatment Rates Among Patients with HIV/HCV Co-infection

**DOI:** 10.1093/ofid/ofad500.1319

**Published:** 2023-11-27

**Authors:** Maximilian D Wegener, Ralph P Brooks, Lisa G Nichols, Merceditas Villanueva

**Affiliations:** Yale University School of Medicine - AIDS Program, New Haven, Connecticut; Yale School of Medicine - AIDS Program, New Haven, Connecticut; Yale University, New Haven, Connecticut; Yale School of Medicine, New Haven, Connecticut

## Abstract

**Background:**

U.S. National Viral Hepatitis strategy advocates Health Departments (HDs) use surveillance-based viral clearance cascades to track progress of HCV care. Cascades are also useful at the clinic level, but creation can be challenging. We developed and piloted a novel Data to Care (D2C) case conferencing tool which merges jurisdictional HD and clinic data, and automatically generates HCV treatment cascades for clinic clients with HIV/HCV co-infection, identifying gaps to be improved.

**Methods:**

Six diverse state/county health departments (HDs) used the excel-based tool with 14 Ryan White-funded clinics to create real-time HCV treatment cascades for clients who received HIV related medical services (1/1/2018–8/31/2021) with a HCV diagnosis (through 11/30/2021). Cascade steps included: HCV PCR testing, treatment eligibility, treatment initiation, and cure (SVR). Barriers to treatment were captured to aid in developing personalized engagement plans. Virtual case conferencing occurred quarterly with HD champions and clinic staff for 1 year. Three models, dependent on HD resources and statutory limitations, were used to identify co-infection: A) HD generated a co-infection list from centralized HIV/HCV surveillance data, B) clinic created a list of persons with HIV that HD matched to HCV surveillance or C) clinic generated a co-infection list from medical records.

**Results:**

For list creation: 1 HD used model A, 2 used model B, 1 used model C, and 2 used a combination of B and C. Among 1249 overall clients with co-infection, 282 (23%) were Black; 842 (67%) were born male. Baseline cascades as of 11/30/2021 showed 102 (8%) needed PCR, 685 (55%) were eligible for HCV treatment and 357 (52% of eligible) achieved SVR. SVR rates varied by clinic. Substance use was a primary barrier to treatment, although most clients experienced multiple barriers.

**Conclusion:**

Our tool uses both surveillance and clinical care data to generate a real-time clinic-specific HCV treatment cascade for clients with HIV/HCV co-infection. It is more granular than using surveillance data alone, enabling clinics in collaboration with jurisdictional HDs to overcome personal barriers to care. Used longitudinally, quarterly case conference cascades allow for insight into progress.

**Disclosures:**

**Ralph P. Brooks, MS**, Merck: Stocks/Bonds

